# Daily Tasks and Willingness to Work of Dental Hygienists in Nursing Facilities Using Japanese Dental Hygienists’ Survey 2019

**DOI:** 10.3390/ijerph18063152

**Published:** 2021-03-18

**Authors:** Yuki Ohara, Yoshiaki Nomura, Yuko Yamamoto, Ayako Okada, Noriyasu Hosoya, Nobuhiro Hanada, Hirohiko Hirano, Noriko Takei

**Affiliations:** 1 Japan Dental Hygienists’ Association, Tokyo 169-0071, Japan; nori@pm-ms.tepm.jp; 2 Research Team for Promoting Independence and Mental Health, Tokyo Metropolitan Institute of Gerontology, Tokyo 173-0015, Japan; h-hiro@gd5.so-net.ne.jp; 3 Department of Translational Research, School of Dental Medicine, Tsurumi University, Yokohama 230-8501, Japan; nomura-y@tsurumi-u.ac.jp (Y.N.); hanada-n@tsurumi-u.ac.jp (N.H.); 4 Department of Endodontology, School of Dental Medicine, Tsurumi University, Yokohama 230-8501, Japan; yamamoto-y@tsurumi-u.ac.jp (Y.Y.); hosoya-n@tsurumi-u.ac.jp (N.H.); 5 Department of Operative Dentistry, School of Dental Medicine, Tsurumi University, Yokohama 230-8501, Japan; okada-a@tsurumi-u.ac.jp; 6 Department of Dental and Oral Surgery, Tokyo Metropolitan Geriatric Hospital, Tokyo 173-0015, Japan

**Keywords:** dental hygienist, nursing facilities, oral hygiene, long-term care, interprofessional collaboration

## Abstract

Oral health care by dental hygienists contributes to the maintenance of nutritional and general health for older people in nursing facilities. This study aimed to investigate daily tasks and willingness to work among dental hygienists working in nursing facilities in Japan. In 2019, using a self-administered questionnaire, a postal cross-sectional survey was conducted among members of the Japanese Dental Hygienists’ Association. Questionnaires were distributed to all 16,722 Association members (responses, *n* = 8932; return rate, 53.4%). We analysed data from 368 dental hygienists currently working in nursing facilities. Item response theory and correspondence analyses were performed. In total, >90% of dental hygienists undertook oral examinations and provided oral hygiene instructions to residents and facility staff. In contrast, the implementation rate of tasks related to interprofessional collaboration was relatively low (approximately 50%), and 72.6% of dental hygienists indicated that they wanted to continue working in nursing facilities. Their willingness to work was closely associated with work involving interprofessional collaboration. Our study findings showed that dental hygienists’ work content in nursing facilities was diverse, but that there was variation in implemented tasks. Willingness to continue working in nursing facilities was associated with interprofessional collaboration among dental hygienists.

## 1. Introduction

In rapidly aging societies, maintaining good oral health status among older adults is a global concern as oral health is closely linked to general health and quality of life [1,2,3]. Particularly, care-dependent older adults living in nursing facilities with poor cognitive function and decreased independence in daily life may also have poor oral health [4,5]. Several studies undertaken in Sweden have reported the importance of ensuring daily oral care by nursing facility staff; however, it is challenging for different professionals to implement oral care because of a lack of guidelines concerning oral care practices and information sharing [6,7,8]. Oral health management and educational interventions by dental professionals for older people requiring nursing care have been emphasised in studies showing these interventions are effective in preventing aspiration pneumonia and in improving nutritional status [9,10,11,12]. Considering these findings, oral hygiene interventions by dental hygienists are likely to help prevent inflammation due to bacterial infections and improve intraoral environments for dietary intake in care-dependent older adults. Therefore, professionally delivered oral care contributes to both an enhanced oral health status and good general health.

The provision of optimal oral health care services for older people in nursing facilities has been a major challenge for countries with an aging population. It has been reported that providing such care for older adults could contribute to the quality of overall care by reducing the burden on caregivers [13]. In this context, a shortage in the nursing staff workforce makes providing oral care challenging due to the inability of staff to spend adequate time to undertake the required care and due to a lack of cooperation from older adult residents [14]. Prompt dental professional involvement is required to resolve difficulties in oral care maintenance. Furthermore, the maintenance of good oral health status in older adults needing long-term care requires collaboration between the care personnel who are the providers of routine oral care and the dental hygienists who are oral health professionals. Japan has a rapidly increasing aging population, and a long-term care insurance system was introduced in 2001 that provides services for older adults requiring long-term care [15]. Within this system, dental hygienists are financially incentivised to provide oral health management to older people requiring long-term care in nursing facilities [16]. This system has enabled dental hygienists to undertake oral hygiene procedures such as removing oral debris, as well as provide oral hygiene guidance to residents and institutional personnel. The number of dental hygienists working at long-term care facilities has recently increased in Japan [17], and the demand for dental hygienists to provide preventive oral hygiene management to residents of nursing facilities is expected to continue to increase in future.

To address the oral health needs of older adults who require nursing, securing sufficient numbers of dental hygienists to act as professional oral health care providers is an urgent issue. To ensure a stable workforce, it is necessary to specify the tasks involved and identify any work challenges dental hygienists encounter to resolve any issues when working in nursing facilities. However, there have been limited studies concerning the current status and challenges facing dental hygienists in nursing facilities. Clarification of the role of dental hygienists in the arena of geriatric caregiving is needed. Every five years, the Japan Dental Hygienists’ Association conducts a survey concerning the working status of dental hygienists [18]. In the latest survey, items concerning daily tasks undertaken in nursing facilities were included. A dental hygienist’s routine tasks in a nursing facility comprise several components; therefore, there is significant information associated with a dental hygienist’s work involving older adults requiring long-term care. Moreover, a multi-disciplinary team approach is needed in a nursing facility; therefore, a dental hygienist is required to work in collaboration with staff from other disciplines. An enhanced understanding of the priorities of dental hygienists’ work in nursing facilities can help clarify the needs of dental hygienists in greater detail. We considered that clarification of a dental hygienist’s role by analysing the nature of their work could improve patient quality of care. Furthermore, in terms of securing human resources for future oral care service providers, it is necessary to determine the relationship between the nature of this work and dental hygienists’ willingness to work. In particular, we assumed that a willingness to continue working would be an important factor associated with undertaking daily tasks. Therefore, this study aimed to assess the willingness of dental hygienists in Japan to work in nursing facilities, and to specify the daily tasks involved in this type of work.

## 2. Materials and Methods

### 2.1. Study Design and Participants

Since 1981, the Japanese Dental Hygienists’ Association has conducted surveys on the employment status of dental hygienists in Japan every five years [18,19,20,21,22]. Our study population was limited to members of the Japan Dental Hygienists’ Association. Questionnaires were posted to all Association members (*n* = 16,722) on 16 October 2019, and members were requested to return the completed questionnaire using a self-addressed pre-paid envelope. By 30 November 2019, 8932 responses had been returned (return rate, 53.4%), which we used for the analysis. Anonymously collected data from 368 actively working dental hygienists in nursing facilities were analysed. This study was conducted in accordance with the Declaration of Helsinki, and was approved by the Ethics Committee of the Tsurumi University School of Dental Medicine (approval No. 1837). Informed written consent was obtained from all participants.

### 2.2. Questionnaire

The questionnaire used in this study, originally designed by the Japanese Dental Hygienists’ Association and by the authors, consisted of 101 items, including demographic factors, employment status, and the nature and value of the work [18,19,20,21,22].

Dental hygienists who indicated that they currently worked in nursing facilities were asked questions concerning their daily tasks using 16 dichotomous items. Regarding their daily tasks at nursing facilities, the participants were requested to answer whether or not they performed the following tasks routinely: (i) an intraoral examination, (ii) planning of oral hygiene management procedures, (iii) oral hygiene instructions for residents, (iv) advice and instructions for facility staff, (v) oral hygiene procedures, (vi) instructions concerning denture maintenance, (vii) topical fluoride application, (viii) evaluation of oral function, (ix) training for oral function improvement, (x) training for swallowing function, (xi) training in daily oral care for facility staff, (xii) coordination with dentists and dental clinics, (xiii) assisting dental check-ups, (xiv) regular participation at conferences related to their occupation, (xv) participation in entrance and exit conferences, and; (xvi) observation of residents’ mealtimes. Concerning their willingness to work, participants were asked: ‘Do you want to continue working in your current job in future?’ with the following response options: ‘yes, ‘no’, or ‘not sure’.

### 2.3. Statistical Analysis

Descriptive statistics for the daily tasks were analysed. We constructed a three-parameter logistic model using item response theory (IRT) analysis to calculate item discrimination, item difficulties, and item guesses in relation to daily tasks [18,19,20,21,22]. The item response and information curves regarding each dental hygienist’s tasks were illustrated graphically. The analyses were performed using R software version 3.5 with the LTR and package ‘irtoys’ using the following formula (Institute for Statistics and Mathematics, Vienna, Austria):

(1) $$P_{i}\left(\theta \right)=\frac{\left(1-c_{i}\right)}{1+e^{-Da_{i}\left(\theta -b_{i}\right)}}$$

where *a_i_*: discrimination, *b_i_*: difficulty, and *c_i_*: guessing.

Cross-tabulation was performed on items related to willingness to work and daily tasks. Correspondence analysis was performed using this cross-tabulation. To visualise the relationships, the results were graphically illustrated as biplots [23,24]. The survey items were determined from previous survey results [19,20,21]. Dichotomous items do not gather in-depth information similar to that acquired using a Likert-type scale. However, using item response analysis, results obtained with dichotomous variables are simple to interpret [19]; therefore, we decided to use dichotomous variables in this study. Detailed and meticulous information, rather than a simple descriptive analysis of frequency, can be obtained using IRT, which is a valuable tool that has been frequently applied in earlier studies [19,20,21]. SPSS Statistics version 25.0 (IBM, Tokyo, Japan) software was used for the analysis.

## 3. Results

### 3.1. Participant Characteristics

**Figure 1:**
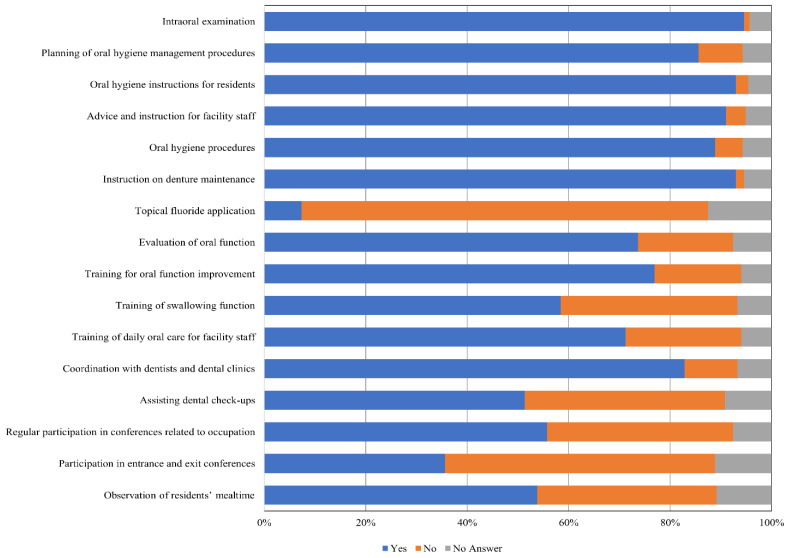
Performance of dental hygienists’ daily tasks when working in nursing facilities. The bar graph shows participants’ responses (in percentages) according to whether each task was performed by a dental hygienist at a nursing facility.

The average participant age was 52.92 ± 9.23 (range, 23–77) years. The average number of years of experience as a licensed dental hygienist was 23.72 ± 10.43 (range, 0–51). The results of descriptive statistics concerning the items specifying the daily tasks undertaken by dental hygienists in nursing facilities are shown in Figure 1. The daily task with the highest implementation rate was an intraoral examination (94.6%), followed by an introduction on denture maintenance and oral hygiene instructions for residents (92.9%), and advice and instructions for facility staff (91.0%). Therefore, >90% of dental hygienists in this study were involved with performing oral hygiene management, including providing instructions for both residents and facility staff. The implementation rate concerning the oral function evaluation was 73.6%, training for oral function improvement was 76.9%, and training for swallowing function was 58.4%. In terms of responses regarding oral function management, implementation rates were lower than those regarding oral hygiene management. Additionally, participation in mealtime observation was 53.8%, and regular participation in conferences related to occupation was 55.7%. Further, the implementation rate concerning daily tasks related to cooperation with multiple disciplines was approximately 50%.

### 3.2. IRT Analysis of Dental Hygienists’ Daily Tasks at Nursing Facilities

**Figure 2:**
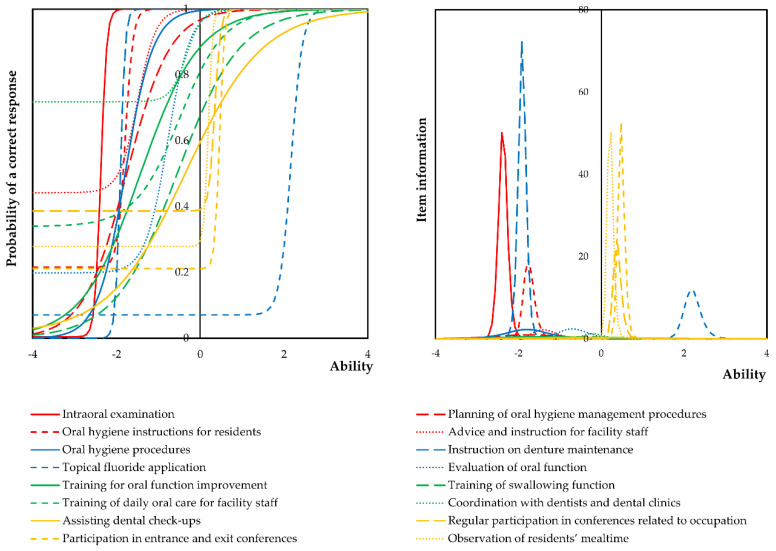
Item response curves and item information curves concerning items for daily tasks at nursing facilities. Ability, expressed as a scale on the *X*-axis, represents the weighted score of the total performance rate. An item response curve or item information curve in a backward direction indicates that the task corresponding to the item was frequently performed. In contrast, an item response curve in a forward direction indicates that the task corresponding to the item was rarely performed. Steep item response curves and high item information curves indicate that, when these tasks were implemented, other tasks were easily performed.

To determine the difficulty and performance of daily tasks, we undertook IRT analysis. Figure 2 shows item response curves and item information curves for the daily tasks of dental hygienists’ undertaken in nursing facilities using a three-parameter logistic model based on IRT. The constructed model is presented in Table S1. The horizontal axis shows the participants′ ability, and the item response curve axis shows a positive response to each item. Discrimination refers to how well an item can distinguish between respondents with low and high abilities. In this case, respondents with high ability levels frequently responded ‘Yes′ to the items, whereas respondents with low ability levels frequently responded ‘No’, with low and flat curves indicating low discrimination. The item response curve shows how precisely each item measures latent traits at various levels. A greater area under this curve indicated that ‘Yes′ was answered for all items at a higher rate, and these item responses may indicate higher levels of job attractiveness among dental hygienists. Of these dental hygienists, most performed tasks associated with oral hygiene management, such as intraoral examination, instructions on denture maintenance, oral hygiene instructions for residents, and advice and instructions for facility staff. Furthermore, the task of ‘coordination with dentists and dental clinics’ was considered to be one that most dental hygienists undertook routinely due to the high frequency of an appropriate response. Conversely, information sharing and collaboration with other medical staff, such as participation in conferences, was challenging to perform. The item information curves were clearly separated and very high; therefore, there were some groups that mainly performed tasks associated with oral hygiene management and active collaboration with other medical professionals.

### 3.3. Correlation between Daily Tasks and a Willingness to Work

**Figure 3:**
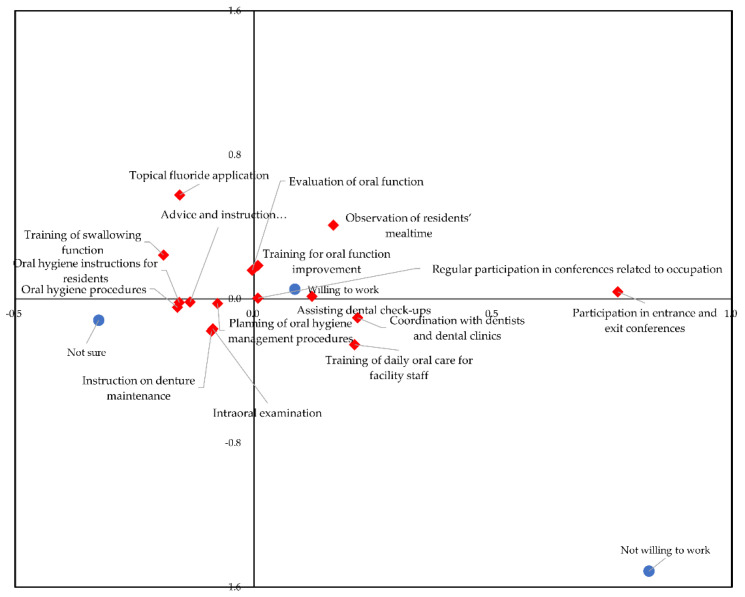
Biplots of willingness to work and daily tasks of dental hygienists at nursing facilities. Blue plots correspond to a willingness to work, and red plots correspond to daily tasks that dental hygienists performed in nursing facilities. Closely located plots are highly coincident.

To investigate the correlation between daily tasks and a willingness to work in nursing facilities, correspondence analysis was undertaken, and the results are shown in Figure 3. Cross tabulations for correspondence analysis are shown in Table S2. Among the participants, 72.6% responded that they would like to continue working in nursing facilities, 22.8% responded, ‘not sure’, and 1.1% responded, ‘not willing to work’. The tasks of assisting dental check-ups, regular participation in conferences related to occupation, and evaluation and training of oral function were more closely associated with a willingness to work than administrative tasks associated with maintenance of oral health status. In contrast, ‘not willing to work’ was not specifically linked to performing daily tasks, indicating that no characteristic daily tasks were associated with an unwillingness to work among dental hygienists. Similar trends were observed for dental hygienists who answered ‘not sure’.

Tasks requiring interprofessional collaboration were closely associated with “willing to work”, indicating that performing tasks of oral hygiene management and jobs requiring interprofessional collaboration correlated with a willingness to work. These results suggest that dental hygienists who were willing to work gave interprofessional collaboration a higher priority among their daily tasks in nursing facilities.

## 4. Discussion

In this study, we investigated daily tasks and willingness to work among dental hygienists working in nursing facilities in Japan. To our knowledge, this is the first report describing detailed characteristics concerning daily tasks performed by dental hygienists in nursing facilities. There are 1282 dental hygienists currently engaged in the care of older adults in Japan [25]. Thus, our study findings in relation to 368 dental hygienists are likely to generally reflect the actual status of dental hygienists working in nursing facilities in Japan [25]. The Japanese insurance system provides coverage to older adults through medical insurance and long-term care insurance. Basic dental care, such as treatment of dental caries and periodontal disease, and prosthodontic procedures are covered by medical insurance, and oral health management for elderly requiring nursing care by dental hygienists is covered by long-term care insurance. Therefore, in Japan, the system for dental service delivery for older adults requiring long-term care is generally well established. Older adults who do not require intensive medical care can reside in nursing facilities. The number of residents in nursing facilities in Japan was reported to be approximately 950,000 in 2019, which was a 1.8-fold increase compared with 10 years previously, and this trend is likely to continue [26]. Therefore, securing human resources concerning dental hygienists to meet the needs of enhancing oral health care for older adults is necessary.

One pioneering study reported that regular oral care by dental professionals reduced the incidence of fever or pneumonia [9], suggesting that an improvement in oral hygiene and function through regular care interventions performed by dental professionals had a positive effect on general health status. Based on that study, the importance of oral care among older adults has been recognised, and nursing facilities in Japan have gradually introduced oral care for their older adult residents. To provide oral health management, dental hygienists from dental clinics regularly visit nursing facilities, as most dental hygienists in Japan work in dental clinics. However, dental care provided by visiting staff once or twice a week has several limitations. Daily oral care is indispensable to maintaining oral health, especially among those who cannot perform oral self-care independently. Therefore, the number of dental hygienists stationed in nursing facilities has gradually increased [17].

When interpreting the descriptive analysis concerning daily tasks, >90% of dental hygienists performed oral hygiene management, including oral hygiene procedures and giving instructions to both residents and facility staff. Moreover, the item response curve showed a backwards shift for most items, except for topical fluoride application, showing that many dental hygienists performed oral hygiene management and training for oral function improvement, and engaged in interprofessional collaboration, such as participating in conferences. This finding suggests that dental hygienists working in nursing facilities are required to carry out a variety of differing daily tasks compared with dental hygienists working in dental clinics [18]. Thus, it would appear that dental hygienists play an important professional role in oral health care in nursing facilities. Lindqvist et al. [7] reported that oral care should be prioritised in nursing facilities. However, oral care is complex for older adults, and nursing facility residents often have difficulty maintaining an adequate level of oral hygiene because of a decreased ability to perform daily self-care, for example, due to the progression of dementia [4,5,27]. Wårdh et al. [28] reported that although nursing staff recognised the importance of oral care, they had low knowledge levels regarding oral care. Continuous theorised oral health education for nursing staff by dental hygienists is required [6,8,27]. Previous studies have shown the effectiveness of professional oral hygiene intervention. Therefore, it is necessary for dental hygienists to engage in long-term care and form part of the staff in nursing facilities to enhance oral hygiene care through specialised care provision and to provide adequate education to nursing staff. While the number of dental hygienists working in nursing facilities is small [17], the increasing aging population warrants a greater number of dental hygienists to maintain the oral health of older adults requiring long-term care.

In this study, IRT analysis indicated that dental hygienists working in nursing facilities performed fewer tasks requiring collaboration with other members of multi-disciplinary teams, such as participation in conferences, compared with performing oral hygiene management tasks, such as oral hygiene procedures and providing training or instructions. Specifically, for dental hygienists stationed in nursing facilities in Japan, oral hygiene management was prioritised above training and interprofessional cooperation in terms of daily tasks. Dental hygienists working in nursing facilities may perceive the maintenance of good oral hygiene for infection prevention, including aspiration pneumonia, as a key component of their work.

Moreover, in the correspondence analysis, items concerning collaboration with other professionals, such as ‘regular participation in conferences’ and ‘observation of residents’ mealtimes’ were closely associated with a ‘willingness to work’, indicating that there was a relationship between willingness to work continuously in nursing facilities and interprofessional collaboration. In other words, interprofessional collaboration was one of the characteristics that respondents felt made working in a nursing facility attractive. It appeared that dental hygienists in tasks that involve an interprofessional approach in nursing facilities might encourage their continued employment and facilitate retaining a stable workforce. Although it was not possible to determine causal relationships due to the study’s cross-sectional design, active involvement in interprofessional collaboration might have a favourable effect on the work of dental hygienists in long-term care facilities. Encouraging dental hygiene participation using an interprofessional approach could be a key factor in improving hygienists’ skill base and their willingness to work at nursing facilities. Interprofessional collaboration should first be implemented at the educational level to help develop trust and understanding of each profession’s role in health care. The World Health Organization recommends training for interprofessional education [29]. Good collaboration among health and social care professionals is essential to enhance quality of care for older adults requiring long-term care [30,31]. Similarly, several previous reports have suggested the importance of dental hygienists’ involvement in interprofessional collaboration [32,33,34,35]. Luebbers et al. reported that most physicians in their study considered that dental hygienists were professionally knowledgeable and played an important role in interprofessional teams [35]. The participation of dental hygienists is likely to provide added value to healthcare and caregiving practice by leading to enhanced understanding of a patient’s health status. However, barriers to interprofessional collaboration, such as a lack of communication and information sharing, have been reported [36]. In our study, factors influencing the low interprofessional collaboration compared with other daily tasks were not identified and further studies are needed to determine specific factors that could enhance dental hygienists’ interprofessional collaboration. We analysed data obtained from dental hygienists’ responses to a self-administered questionnaire; therefore, it is not known how personnel from other occupations, including caregiving personnel and nurses, consider the role of dental hygienists in nursing facilities. It may be necessary to investigate their perceptions of each other’s occupations to promote better interprofessional collaboration.

A further issue has arisen recently concerning the psychological effects of the COVID-19 pandemic on dental hygienists. It is likely that the psychological burden on dental hygienists working in nursing facilities, who are in particularly close contact with older adults at an increased risk of COVID-19 infection, is substantial. Further studies will be necessary to evaluate this issue specifically [37].

This study had some limitations. First, varying participant backgrounds may have influenced the results, such as years of education prior to obtaining a dental hygienist′s license and years of clinical experience, which may have led to differences in a willingness to work. Second, the duties of dental hygienists are stipulated and regulated through specific registration bodies in each country; therefore, the types of tasks may vary widely, limiting the generalisability of our study findings outside Japan. However, this study identified key findings in regard to the types of tasks performed and the willingness to work as a dental hygienist, and our results highlight the potential to expand dental hygiene work as oral professionals in the field of caregiving. These findings could help to refine public health policies in response to rapidly aging societies, which are presently of global concern. Currently, most dental hygienists work in private dental clinics, but the role of dental hygienists is anticipated to comprise interprofessional collaboration in future. Further studies to determine how to ensure a stable dental hygienist workforce, particularly in relation to dental hygienists working in nursing facilities, are needed.

## 5. Conclusions

Japanese dental hygienists working at nursing facilities were found to undertake diverse daily tasks and to highly prioritise oral hygiene management-related procedures and guidance; however, they gave low priority to interprofessional collaboration, although it was closely associated with a willingness to work. Work content also appeared to be diverse, but there was variation in implemented tasks. It would appear that improving interprofessional collaboration among dental hygienists could be a factor in promoting a willingness to continue working in nursing facilities.

## Data Availability

The data of the present study were used under license for the current study and, therefore, are not publicly available.

## Supplementary Materials

The following are available online at https://www.mdpi.com/1660-4601/18/6/3152/s1, Table S1: Three-parameter logistic model based on item response theory of tasks of dental hygienists in nursing facilities, Table S2: Comparison of tasks of dental hygienists in nursing facilities and willingness to work.
