# Supplementary material for: Daily Tasks and Willingness to Work of Dental Hygienists in Nursing Facilities Using Japanese Dental Hygienists’ Survey 2019
Source: Int J Environ Res Public Health. 2021 Mar 18;18(6):3152. doi: 10.3390/ijerph18063152 (PMC8003160; doi:10.3390/ijerph18063152)
Supplement: Supplementary file 1 [file ijerph-18-03152-s001.pdf]

Table S1. Three parameter logistic model based on item response theory of dental hygiene practice

	Discrimination	Difficulty	Guessing
Intraoral examination	14.47	-2.38	0.00
Planning of oral hygiene management procedures	1.94	-1.76	0.00
Oral hygiene instructions for residents	10.85	-1.79	0.22
Advice and instruction for facility staff	4.62	-1.49	0.44
Oral hygiene procedures	3.01	-1.79	0.00
Instruction on denture maintenance	17.10	-1.91	0.00
Topical fluoride application	7.43	2.17	0.07
Evaluation of oral function	3.75	-0.78	0.20
Training for oral function improvement	1.43	-1.43	0.00
Training of swallowing function	1.34	-0.55	0.00
Training of daily oral care for facility staff	1.87	-0.49	0.34
Coordination with dentists and dental clinics	5.24	-0.33	0.72
Assisting dental check-ups	1.09	-0.34	0.01
Regular participation in conferences related to occupation	14.37	0.34	0.39
Participation in entrance and exit conferences	17.80	0.46	0.21
Observation of residents’ mealtime	19.76	0.19	0.28

Table S2. Comparison of dental hygiene practice between willingness to work

	Willing to work (n=267)		Not willing to work (n=4)		Neither (n=84)		*p*-value*
	n	%	n	%	n	%	
Intraoral examination	260	97.4	4	100.0	82	97.6	0.517
Planning of oral hygiene management procedures	238	89.1	3	75.0	73	86.9	0.825
Oral hygiene instructions for residents	256	95.9	3	75.0	81	96.4	0.013
Advice and instruction for facility staff	252	94.4	3	75.0	79	94.0	0.079
Oral hygiene procedures	245	91.8	3	75.0	78	92.9	0.224
Instruction on denture maintenance	256	95.9	4	100.0	81	96.4	0.883
Topical fluoride application	21	7.9	0	0.0	6	7.1	0.820
Evaluation of oral function	208	77.9	2	50.0	60	71.4	0.132
Training for oral function improvement	217	81.3	2	50.0	62	73.8	0.124
Training of swallowing function	163	61.0	1	25.0	50	59.5	0.284
Training of daily oral care for facility staff	201	75.3	4	100.0	57	67.9	0.358
Coordination with dentists and dental clinics	236	88.4	4	100.0	65	77.4	0.053
Assisting dental check-ups	146	54.7	2	50.0	41	48.8	0.739
Regular participation in conferences related to occupation	156	58.4	2	50.0	46	54.8	0.819
Participation in entrance and exit conferences	106	39.7	2	50.0	22	26.2	0.149
Observation of residents' mealtime	156	58.4	1	25.0	40	47.6	0.116

* Chi-square test
